# Evolution of *Peromyscus* *leucopus* Mice in Response to a Captive Environment

**DOI:** 10.1371/journal.pone.0072452

**Published:** 2013-08-06

**Authors:** Robert C. Lacy, Glen Alaks, Allison Walsh

**Affiliations:** Chicago Zoological Society, Brookfield, Illinois, United States of America; National Institute of Allergy and Infectious Diseases, United States of America

## Abstract

Many wildlife species are propagated in captivity as models for behavioral, physiological, and genetic research or to provide assurance populations to protect threatened species. However, very little is known about how animals evolve in the novel environment of captivity. The histories of most laboratory strains are poorly documented, and protected populations of wildlife species are usually too small and too short-term to allow robust statistical analysis. To document the evolutionary change in captive breeding programs, we monitored reproduction and behavior across 18 generations in six experimental populations of *Peromyscus* *leucopus* mice started from a common set of 20 wild-caught founders. The mice were propagated under three breeding protocols: a strategy to retain maximal genetic diversity, artificial selection against stereotypic behaviors that were hypothesized to reflect poor adaptation to captivity, and random bred controls. Two replicates were maintained with each protocol, and inter-replicate crosses at generations 19 and 20 were used to reverse accumulated inbreeding. We found that one of the stereotypic behaviors (repetitive flipping) was positively associated with reproductive fitness, while the other (gnawing) was relatively invariant. Selection to reduce these stereotypic behaviors caused marked reduction in reproduction, and populations not under artificial selection to reduce these behaviors responded with large increases in flipping. In non-selected populations, there was rapid evolution toward much higher proportion of pairs breeding and more rapid conception. Litter size, pup survival, and weaning mass all declined slowly, to the extent that would be predicted based on inbreeding depression. Inter-crossing between replicate populations reversed these declines in fitness components but did not reverse the changes in behavior or the accelerated breeding. These findings indicate that adaptation to captivity can be rapid, affecting reproductive patterns and behaviors, even under breeding protocols designed to minimize the rate of genetic change due to random drift and inadvertent selection.

## Introduction

Darwin [1] noted that morphological and behavioral traits evolve when species are propagated in captivity, as the result of artificial selection for domestication and other traits favored by humans, natural selection for adaptation to the captive environments, and relaxed selection for traits important only in the wild. The changes that occur in wildlife species during generations of captive breeding have implications for evolutionary biology, for the use of laboratory stocks as model organisms for comparative, evolutionary, and mechanistic studies, for the process of domestication of species for human use [2], and for conservation. Recent studies of populations in modified wild habitats have indicated that rates of evolution can be faster than expected [3–5], and rates of change in captive environments can be several-fold greater [6,7].

Long-term laboratory stocks, often with poorly known origin and histories, have been used for decades for studies of behavior, genetic control of morphological, physiological, and behavioral traits, and mechanisms of disease resistance. However, very little is known about how the populations have changed due to selection, genetic drift, or inbreeding during the generations of captive breeding. With respect to species conservation, increasingly captive breeding has been used as a means to secure the future of species that cannot currently be protected adequately in wild habitats, until such time as the wild populations can be reinforced or reestablished from releases [8,9]. However, the genetic changes that occur in captivity can create animals that are poorly suited for life in the wild, and may even be damaging to species conservation if they interbreed with remnant wild populations [6,10]. While a number of populations have been successfully reestablished in the wild from captive stocks [11–13], many such attempts fail, and translocations of animals from other wild populations are more often successful than the release of captive-born animals [14]. It is therefore important to know what changes are likely to occur in wildlife populations during generations of captive breeding.

Frankham [8] and Williams and Hoffman [15] reviewed the literature on adaptation to captivity, citing studies that reported large and rapid changes. Often, the adaptations to captivity were reflected in faster breeding and increased fecundity. However, most studies reporting rapid reproductive adaptations to captivity have involved fish, insects, or plants, and almost all involved species with high fecundity (e.g., salmon and *Drosophila* ) such that very strong selection coefficients can be imposed. A number of studies have reported morphological changes in captive populations of mammals [16].

Evolution of behavioral traits might be especially likely in the highly modified environment of captivity, with animals facing no need to avoid predation, no competition for resources, and usually no competition for mates. A few studies have reported increases in docility (lack of fearfulness of humans), although often that trait was under direct artificial selection in the captive breeding program (e.g., [17]) or could have been selected inadvertently in the handling protocols (e.g., [18]). Some studies have reported increases in variation in traits for which selection would have been relaxed in captivity, such as anti-predator behaviors [19]. However, in general it is not easy to determine what behaviors in captivity reflect adaptive responses to the novel environment, rather than maladaptive reactions to environments in which the species had not evolved.

Breeding programs for wildlife species are often designed to minimize the rate of genetic change [20,21]. The protocol for selecting breeders that is most effective at retaining genetic diversity is to prioritize animals for breeding based on each animal’s mean kinship to the population [22–24]. This strategy maintains alleles originally contributed by the founders as equally represented as possible by preferentially breeding those individuals that have fewest kin in each generation. It thereby should minimize the rate of adaptation to the immediate environment by removing much of the between-family component of selection. For this reason, it has been recommended that the mean kinship strategy be employed as a means to slow adaptation to captivity in species conservation breeding programs [8,25]. However, the effect of breeding protocols on adaptation to captivity has received little experimental testing [15], with the primary exceptions being studies on *Drosophila* by Frankham and colleagues [8,26–29].

We report here the effects of 20 generations of laboratory breeding of *Peromyscus* *leucopus* mice. Two replicate populations were propagated under each of three protocols for the selection of breeders: (1) random breeding; (2) pairs selected to maximally retain gene diversity; and (3) selection for mice that appeared to be most docile, having the lowest scores for two stereotypic behaviors (“flipping” and “gnawing”) that are common in small rodents in captivity [30]. The replicate populations of each of the first two breeding protocols were crossed during the last two generations of the study, in order to determine if changes in traits could have been due solely to accumulated inbreeding. We describe here the observed effects of the generations in captivity on the docility behaviors and measures of reproductive performance. Collaborators will report in further papers on the changes observed in morphology, molecular genetic assays of variation, quantitative genetic partitioning of genetic control of behavioral and morphological traits, and corticosteroid hormones that respond to stress.

## Methods

### Ethics statement

The study protocols were approved by the Institutional Animal Care and Use Committee of the Chicago Zoological Society. Mice were collected under Scientific Permit W01.0845 from the Illinois Department of Natural Resources.

### Subjects

The founders for the laboratory populations of *Peromyscus* *leucopus* mice were collected 2-9 October 2001 from the Volo Bog State Natural Area, Lake County, Illinois, USA. The habitat is mature oak woodland and is one of the few remaining representations of the pre-European settlement habitat of that region. Mice were captured in Sherman traps baited with peanut butter, and taken to the research facilities of the Chicago Zoological Society (Brookfield, Illinois). Subsequent mark-recapture studies indicate that the resident population of *Peromyscus* *leucopus* in the 4 ha area that was trapped is about 200 mice at that time of the year. The area trapped is contiguous with extensive areas of habitat suitable for the species. From 59 *P* *. leucopus* that were trapped, 19 pairings were made in the laboratory (other mice were sacrificed for disease testing or used in other studies), and 12 of the pairs successfully reproduced. The breeding pairs were left together for five to 10 months, during which time they produced up to 10 litters. The 10 most prolific pairs, each producing five or more litters, were used as the founders for this study.

Progeny of each founder pair were allocated equally among six experimental populations, with three to five (usually four) progeny assigned from each founder pair to each experimental population, so that each experimental population started with a total of 40 offspring from the 10 pairs of wild-caught founders, with the same proportional representation of founder pairs across the experimental populations. These six populations were then randomly assigned to three breeding protocols with two replicate populations per protocol.

Laboratory stocks were housed in a common room, maintained at a mean temperature of 21 C, under a 14: 10 L:D photoperiod. Mice were housed in 18 cm x 28.5 cm x 12.5 cm deep clear plastic cages, provided with corn cob bedding, and *ad libitum* water and Agway Prolab RMH2000 Laboratory Animal Diet. Mice selected for experimental breeding were between 70 and 180 days of age at the time of pairing. On the day of parturition, the pups were counted, and the pairs were provided with cotton for constructing nests. Litters were weaned at 20 d of age, at which time they were weighed (to 0.1 g), sexed, ear-punched with identifying codes, and housed three per cage with other newly weaned mice of the same sex. Mice were not handled until they were weaned; thereafter they were handled about once a week for transfer to clean cages. At the time of weaning of the first litter produced by a pair, the sire and dam were separated. Most pairs had bred following the birth of the first litter, and produced second litters within 22 d after weaning the first litter. Thus, each pair could produce at most two litters. Pairs that had not produced a litter were separated 70 d after pairing.

### Behavioral measurements

Between 70 and 80 d of age, each mouse was housed singly for one night for the purpose of observing two behaviors that appear to be stereotypies engaged in by many of the mice during the active, night-time period. One behavior, scored as “Flipping”, consists of repetitive circular flips off the wire top of the cage. This is the stereotypic behavior described as “back-flipping” and seen in a number of small rodents [30], including *Peromyscus* [31]. The other behavior, scored as “Gnawing”, consists of gnawing at the cage top bars. This “bar-mouthing” was described by Würbel [30] as “the main stereotypy in mice” (presumably referring to laboratory *Mus musculus*). Each mouse to be scored for these behaviors (up to 7 mice per night recorded simultaneously) was placed into an individual observation cage with red light illumination, fresh bedding, food, and water during the day (before 5 PM, within the light cycle that had lights off 9 PM to 7 AM), and then the behaviors of the mice were taped using a timer-controlled low-light sensitive, video camera.

The two behaviors were later scored by a single researcher (GA) for each mouse from the video tapes using focal animal sampling in which the duration of each bout of behavior was recorded during a period of 5 minutes between 10 and 11 PM and again four hours later for 5 minutes between 2 and 3 AM. For each of the two behaviors, the score for each mouse was the total time spent in that behavior during the 10 min of observation. Scores ranged from 0 (inactive mice) to 600 s (mice that sustained one of the behaviors continuously during the two 5 minute blocks).

### Pair selection protocols

Mice to be paired for breeding were selected after all mice from their generation of an experimental stock were scored for the Flipping and Gnawing behaviors. The three breeding protocols (see below) were designated as RAN, MK, and DOC, with the replicate stocks designated as RAN1, RAN2, MK1, MK2, DOC 1, and DOC 2. Generations were kept discrete. We designated the generations of experimental breeding by labeling the mice that were laboratory-born offspring of the wild-caught founders as G0, with subsequent generations as G1 through G20. Reproductive performance across the generations is labeled by the generation number of the offspring, so that measurements of reproduction by G0 sires and dams, producing G1 offspring, are labeled as G1 reproduction. Analyses of changes in Flipping and Gnawing across generations began with the G0 captive-born mice, and analyses of reproductive performance began with the G1 pairings, so that scores on wild-caught mice were not included in statistical analyses.

For each pair selection protocol, for each of the two replicate populations, 20 pairs of mice were established each generation, except in a few cases in which fewer than 20 males or 20 females had been produced from the prior generation of breeding. This shortage of mice for breeding caused each of the RAN2, MK1, MK2, and DOC 1 stocks to have fewer than the desired number of pairs in a few of the generations. The DOC 2 had poor breeding performance starting in G5, causing that stock to have only 9 pairs at G6, 17 pairs at G7, 13 pairs at G8, and two pairs at G9. The two pairs of DOC 2 mice at G9 both failed to produce litters.

Inbreeding coefficients of all animals and kinship coefficients between all possible pairs were calculated under the assumption that the wild-caught founders were unrelated and non-inbred. The methods of selection of dams and sires for pairs were as follows: For the RAN protocol, a computer program generated randomly sorted lists of males and females each generation. Pairs were created with the constraint that no pair was accepted if the kinship between the male and female was greater than the mean of all pairwise kinships for that stock at that generation. The coefficient of kinship of a pair is the same as the inbreeding coefficient of any progeny that they produce, so this constraint avoided producing litters that would have inbreeding coefficients more than the average inbreeding that would have been obtained if pairing was completely random. If a pair was rejected because of high kinship, the female was paired instead with the next available mouse on the list of males.

The MK protocol follows methods used to maximally retain gene diversity through minimizing the mean kinship of the selected breeders [22–24,32]. The MK of each individual in a population is the mean of the pairwise kinships of that animal to all animals currently in the population (i.e., in the same generation of the same stock of this study). It is possible that the set of pairs that individually have low mean kinships might be interrelated and therefore collectively not retain as much gene diversity as would a different set of pairs. Therefore, to obtain the set of pairs that collectively have the lowest mean kinship, a method of backwards elimination of least desirable breeders was used. First, from the sex that was in excess, the animal with the highest mean kinship was removed. The kinships of that animal were removed from the population kinship matrix, and mean kinships for all remaining animals were recalculated. This process was repeated until there was a balanced sex ratio among the remaining potential breeders. Then, animals with the highest mean kinships were removed, with the recalculation of mean kinships each time, alternating between a male and a female until there were no animals remaining in the set. The last male and the last female removed in this process were the first pair to be selected as breeders, under the same constraint of avoiding close inbreeding as described above for RAN. The second to last male and second to last female removed in the backward elimination were the second pair selected, and on through the 20 desired pairs. Ivy and Lacy [33] demonstrated that this method selects a set of breeders that retain as much gene diversity as can be obtained by an optimization search across all possible sets of pairs.

The DOC protocol was an artificial selection scheme designed to maximize “docility” through selection against gnawing and flipping. Each generation, the males and females that had the lowest sum of Gnawing plus Flipping scores were selected as breeders, with the lowest scoring male paired with the lowest scoring female, and on through the selection of 20 pairs, with the constraint that no pairs were created that had a kinship greater than the population mean. It should be noted that after G5, this selection scheme had little meaning in the DOC 2 stock, because every available pair was used (although to the extent that there was an excess of either sex, the few animals not used in pairings were those with the highest Gnawing + Flipping). In G14 and G18, the DOC 1 stock did not produce enough mice to allow 20 pairs to be created, so that no selection on behavior was imposed in those generations, as all available DOC 1 mice were used to propagate the population. In both DOC 1 and DOC 2, by G5 there was almost no variation in Flipping, as the behavior had become almost absent, and the subsequent selection was therefore based almost entirely on the Gnawing scores.

### Intercrosses between replicates

In order to determine if the changes observed through the first 18 generations of captive breeding could have been caused by the accumulation of inbreeding in the closed populations, at G19 and G20 crosses were performed between the two replicate populations of each of RAN and MK protocols. (The earlier failure of the DOC 2 population precluded crosses between the DOC replicates.) In G19, 10 pairs were created from each of RAN1, RAN2, MK1, and MK2 – following the same protocol as the prior 18 generations and thereby continuing the accumulation of inbreeding within the populations. To create out-crossed populations for comparison, 10 pairs were established for each of RAN1 x RAN2, RAN2 x RAN1, MK1 x MK2, and MK2 x MK1 male x female crosses. These first generation intercrosses were labeled as RANx1 and MKx1, with reciprocal crosses pooled for analysis. In G20, these crosses between replicates were repeated, creating 20 more RANx1 and 20 more MKx1 pairings. Another 20 pairs were created by crossing RANx1 x RANx1 (to create a RANx2 second intercross generation), and 20 pairs were created by crossing MKx1 x MKx1 (to create MKx2).

### Measures of reproductive performance

Several aspects of reproductive performance were monitored: the success or failure of a pair to produce a litter conceived within the up to 70 d that each pair was kept together (Pr[breed]); the days between pairing and the birth of the first litter (Delay); the number of pups born in each litter (Litter Size); the mass of pups at weaning (Weaning Mass); and the survival of pups from birth to weaning (Litter Survival). The distribution of survival rates of pups in litters is bimodal because often entire litters survive or die. Therefore, for statistical tests, Litter Survival was analyzed as a dichotomous variable, assigned a value of 1 if more than half of the pups in a litter survived to weaning and 0 otherwise. The pups within a litter are often of similar mass at weaning, likely reflecting the maternal care provided more than any characteristic inherent in the pups themselves. Weaning Mass was therefore scored as the mean for each litter, to avoid statistical problems caused by non-independence of littermates. To evaluate the combined effects of fertility, fecundity, pup survival, and growth, the overall reproductive success (RS) was assessed as the total mass of offspring weaned for the zero, one, or two litters produced by each pair.

### Statistical analyses

Throughout this paper, Protocol, Replicate, Generation, and other terms will be capitalized when referring to factors or variables in statistical models. Trends across generations and differences between experimental populations for the continuous variables (Flipping, Gnawing, Delay, Litter Size, Weaning Mass, and RS) were analyzed with ANOVA from General Linear Models. For dichotomous response variables (Pr[breed] and Litter Survival), changes across generations and differences among populations were assessed with logistic regressions fitted with Maximum Likelihood Estimation. All statistical analyses were conducted with SYSTAT version 11 (SYSTAT Software, Inc, Chicago, Illinois).

For variables describing litter traits, there were large differences between first and second litters produced by a pair (mean Litter Size = 4.11 + 0.03 SE and 5.28 + 0.04, mean Litter Survival = 0.798 + 0.009 and 0.915 + 0.006, and mean Weaning Mass = 10.40 + 0.06 and 10.64 + 0.05 for the first and second litters). Therefore, for analyses of these variables, the litter number (1 or 2) was included as a categorical covariate in the models. For variables describing activity behaviors, there were small but significant differences between males and females (Gnawing = 70.3 + 1.6 SE and 63.1 + 1.6, and Flipping = 172.8 + 2.8 and 163.8 + 2.9, for males and females). Therefore, in analyses of these variables, sex was included as a covariate.

Changes across generations in the measured traits could be caused by accumulated inbreeding, or by adaptations to the laboratory environment, or by correlated responses to adaptive changes in traits with which the measured traits have a genetic correlation, or by random genetic drift. Effects of adaptation to the novel environment would be reflected in overall trends across generations, although with possible differences between the breeding protocols because the system of breeding could influence the effectiveness of natural selection. Differences between Protocols that are greater than the differences between Replicates would indicate that the breeding system affected selection or that the behaviors under selection in the Docility protocol affected also the other measured traits. Effects of random drift would be reflected in differences between Replicates within each Protocol.

For ANOVAs of continuous variables, the main effects of Protocol and Replicate within Protocol (designated Replicate{Protocol}) were included in all models, so that tests of differences between trends across generations reflect changes in slope and not any differences between intercepts. Models were run with the effect of (a) Generation (with 1 d.f.), (b) Generation plus a Generation*Protocol interaction (with 2 d.f.), and (c) Generation plus Generation*Protocol-Replicate (with 5 d.f.). The overall effects of Generation were tested with F-tests of the Generation MS (mean square), from model (a) vs the residual (error) MS from the full model (c) that removed all Protocol and Replicate effects. The differences between Protocols was tested with F-tests of the Generation*Protocol MS from model (b) vs the Generation*Replicate{Protocol} MS, in which Generation*Replicate{Protocol} is the Generation*Replicate effect from model (c) nested within Generation*Protocol (resulting in 3 d.f. for the nested effect). Differences between Replicates within Protocols for the slopes across Generations were assessed with F-tests of the Generation*Replicate{Protocol} MS vs the residual MS from the full model (c).

For the dichotomous response variables, changes across generations were assessed with logistic regressions. Significance of a predictor was determined by testing as a chi-square the G-test value of two times the difference between the log-likelihood for the model including the predictor variable and the model excluding it [34]. Models were run with: (a) only main effects of Protocol and Replicate within Protocol, (b) those main effects plus Generation, (c) the main effects, Generation, and Generation*Protocol, and (d) the main effects, Generation, and Generation*Protocol-Replicate. The overall effect of Generation was assessed from model (b)-(a), differences between Protocols were assessed from (c)-(b), and differences between Replicates within Protocols were assessed from (d)-(c).

To examine the direction and magnitude of the differences among slopes, regressions of each trait were calculated for the overall effect of Generation (with effects of covariates Litter Number or Sex removed, when appropriate and as described above), the effect of Generation for each Protocol analyzed separately (across both Replicates), and the effect of Generation for each Replicate analyzed separately. Slopes from linear regressions were used for the continuous traits. For each dichotomous trait, the trend was represented by the change in the response variable from G1 to G2 predicted from the best-fit logistic regression equation. The logistic regression is not linear (although it is nearly so over the range of small measured effects), so the change will not be constant across the range of Generations observed.

To assess the effects of inter-replicate crosses in G19 and G20, for each of the RAN and MK breeding protocols, ANOVAs or logistic regressions (for dichotomous traits) were used to test for differences between the means from the last two generations (G18 and G19) of each replicate (e.g., RAN1, RAN2), the first inter-replicate cross (e.g., RANx1 in G19 and G20) and the second generation cross (e.g., RANx2 in G20). The last two generations of the replicates were combined for these comparisons, because no significant differences were observed between G18 and G19 in any population.

## Results

Table 1 shows the total numbers of pairings, litters produced, pups born, and pups surviving to weaning, and the maximum inbreeding coefficient that accumulated through the 18 generations of laboratory breeding for each Replicate of each Protocol, before the final two generations in which replicates were crossed. Effective population size (N_e_) for each replicate that persisted was estimated from the relationship between N_e_ and the loss of gene diversity: GD_t_/GD_0_ = (1 - 1/(2N_e_))^t^, in which GD_t_ / GD_0_ is the proportional gene diversity after t generations, with GD calculated as the mean of all pairwise kinships within a population [35]. The RAN and DOC protocols resulted in N_e_ less than the 40 mice paired each generation, as a result of some failed pairings and unequal family sizes. The MK protocol, by keeping the contributions of each founder lineage as equal as possible, resulted in N_e_ / N ratios of about 1, and even greater than 1 in MK2 indicating that variation in the contributions of each lineage was less than expected under random breeding of 20 pairs.

**Table 1:** Sample sizes across generations 1 through 18, in each of 6 experimental populations of *Peromyscus* *leucopus* mice, with the maximum level of inbreeding (F) accumulated in each population and the effective population size (N_e_) over the 18 generations.

**Protocol**	**Replicate**	**#Pairs**	**#Litters**	**#Born**	**#Weaned**	**max F**	**N_e_**
Random	RAN1	360	449	1955	1746	0.273	27.7
	RAN2	359	509	2562	2111	0.274	28.8
MK	MK1	352	504	2435	2129	0.227	37.1
	MK2	355	517	2290	1991	0.200	44.2
Docility	DOC 1	356	402	1797	1554	0.295	24.0
	DOC 2^a^	141	95	444	416	0.249	

^a^ The DOC 2 population was maintained only to generation 9, due to reproductive failure.

**Figure 1:**
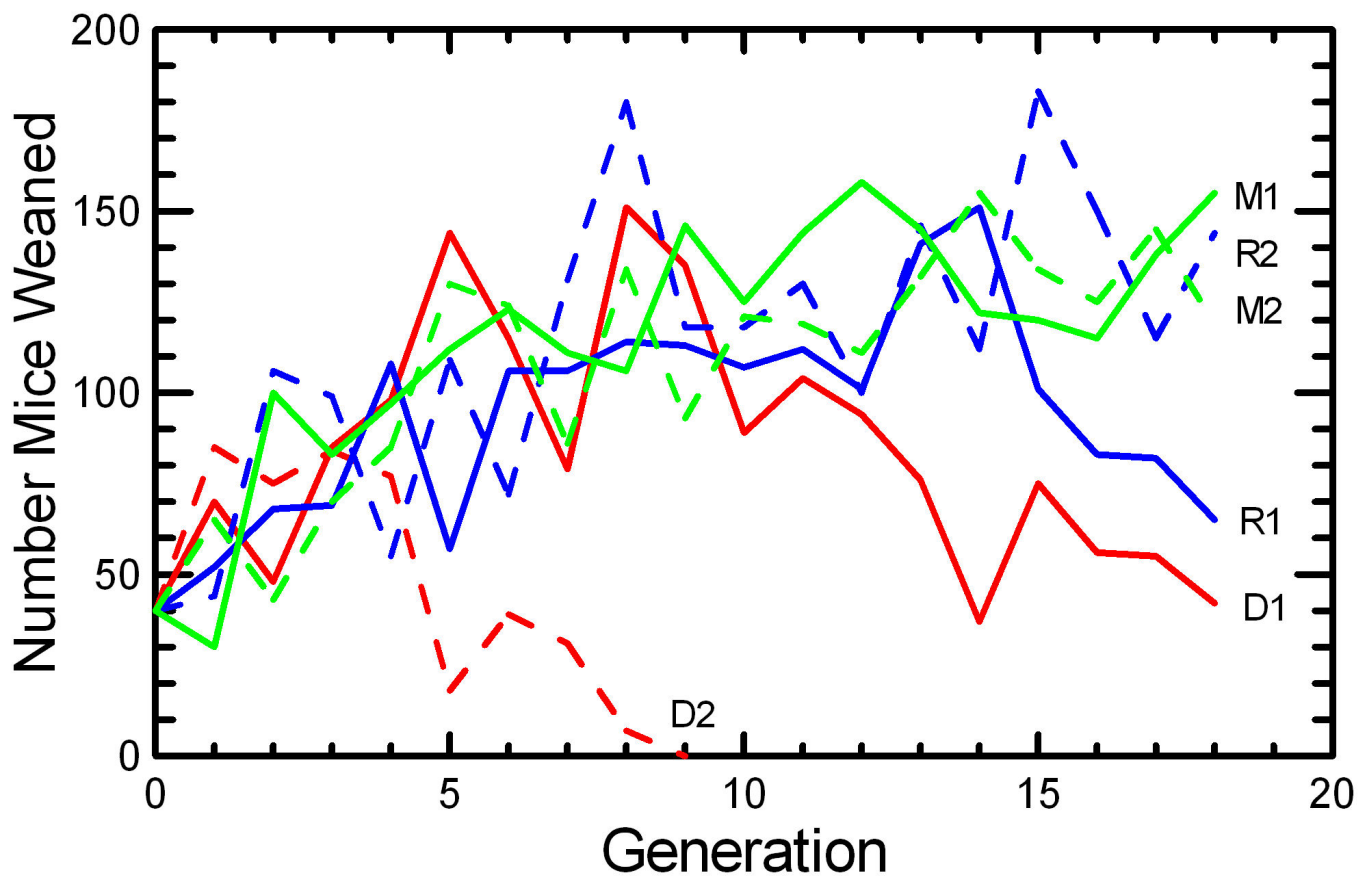
Size of experimental populations of *Peromyscus* *leucopus* mice across generations. Each population started with 40 mice in 20 pairs, and thereafter the population size is the number of mice weaned from up to 20 pairs established each generation. For all figures, the mean values at each generation for RAN replicates are shown in blue, MK replicates shown in green, and DOC replicates shown in red. Lines are labeled as to replicate to the right of each line.

**Figure 2:**
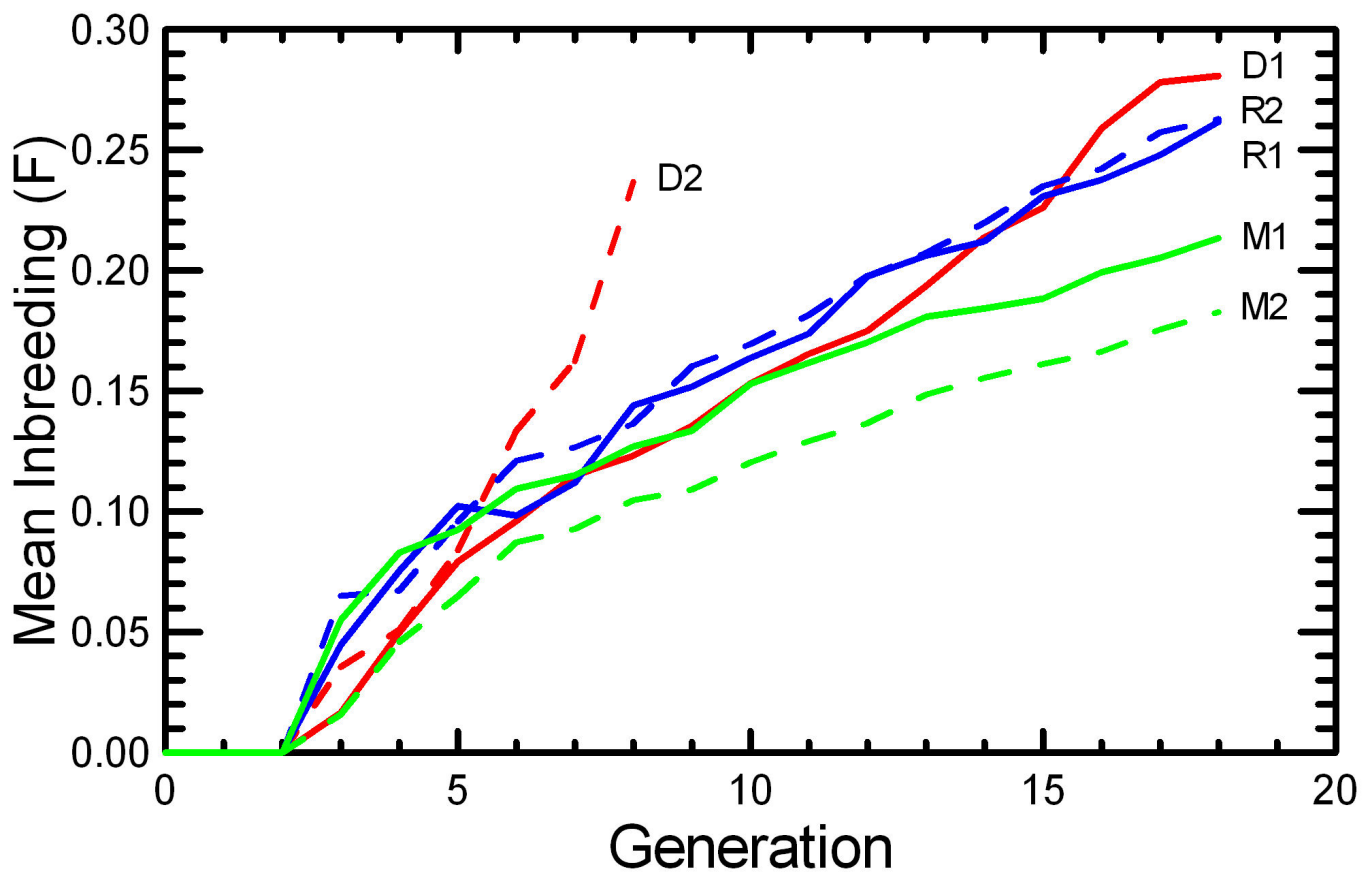
Accumulated inbreeding under three breeding protocols. The mean inbreeding coefficient (F) at each generation accumulated in closed populations of 20 pairs per generation is given for the two replicates of RAN (blue), MK (green), and DOC (red) experimental populations.

Most of the experimental populations had an increasing number of pups weaned across the generations (Figure 1). The growth of experimental populations was due to improved reproductive performance per pair, as a constant number of 20 pairs were established each generation except in the few cases when there were not enough males and females produced the prior generation for all the planned matings. The DOC 2 stock was sustained only through G9, and with fewer than the designed number of pairings from G6 onwards. The loss of the DOC 2 stock occurred as a consequence of rapidly diminishing reproductive success (see below) that was concurrent with rising levels of inbreeding in that stock. Although all populations accumulated inbreeding, the MK strategy slowed inbreeding (due to greater N_e_) relative to RAN, and the DOC protocol led to more rapid inbreeding because of the selection on behavioral traits further reduced N_e_ (Figure 2). As the number of mice available for breeding in DOC 2 declined below 20 pairs per generation, inbreeding accumulated more rapidly. The decline may have been accelerated by inbreeding depression, as fewer of the pairs that were established in the later generations produced any offspring. A similar rise in the rate of inbreeding, decrease in breeding success, and declining population size appeared to be underway in the last few generations of DOC 1.

### Effectiveness of selection for docility

**Figure 3:**
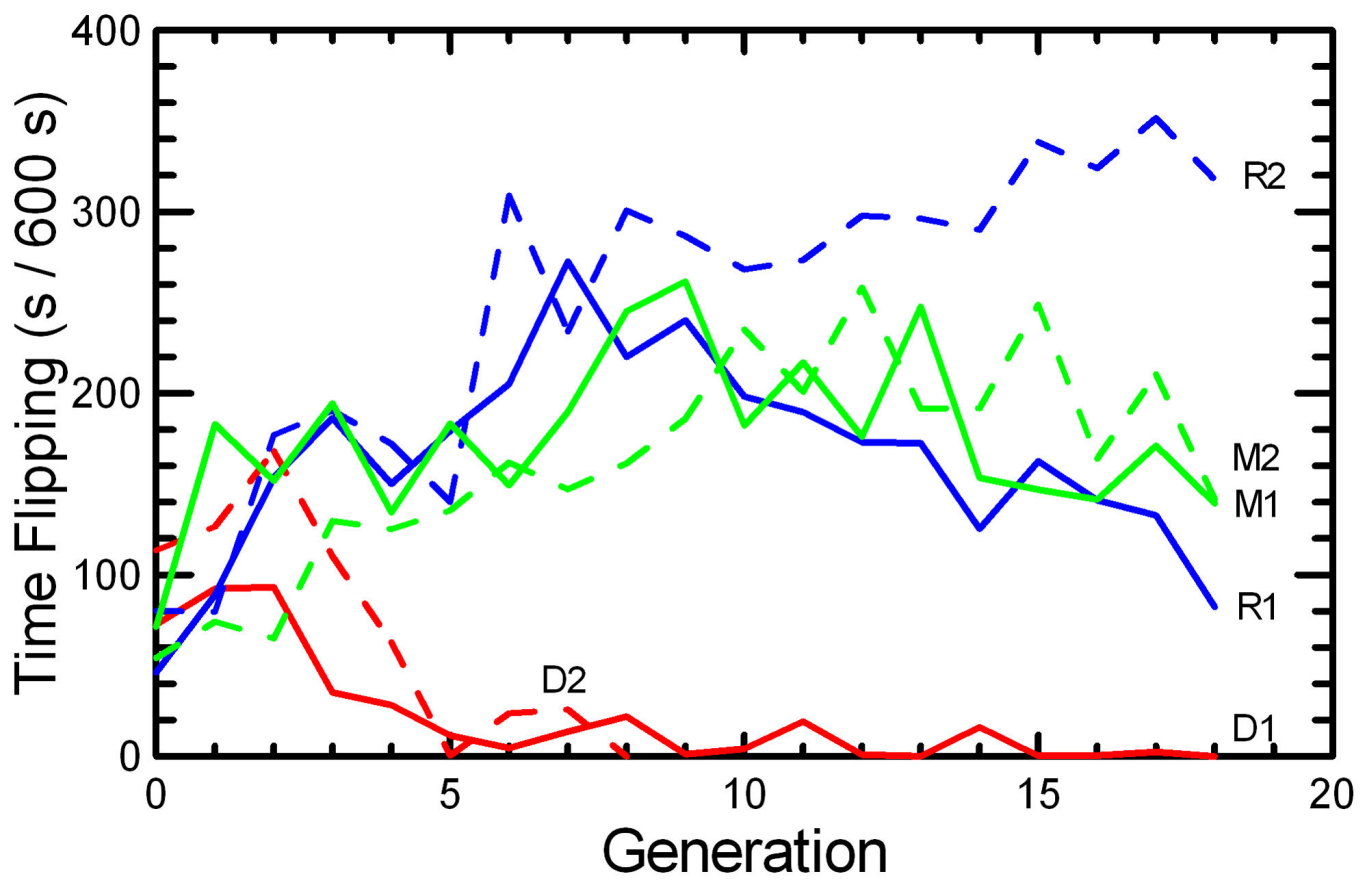
Mean time spent in stereotypic Flipping behavior. The mean number of seconds per 600 s observation period that mice spent in Flipping behavior is given for the two replicates of RAN (blue), MK (green), and DOC (red) experimental populations.

First generation laboratory born mice spent an average of 10% and 13% of the observation time Gnawing and Flipping, respectively, with a wide range among individual mice, from 0 to 96% and 0 to 99%. The behavior scores on wild-caught mice are not strictly comparable to laboratory-born mice, because the past environments of the mice are very different. However, it is notable that wild-caught mice that had not been paired for breeding in the laboratory (n = 14, scored after having been in captivity for 294-300 d subsequent to capture as adults or weaned juveniles) spent an average of 10% of the time Gnawing (range 0 to 45%), but only 0.2% of the time Flipping (with one mouse flipping 3% of the time) when housed in laboratory cages. This confirms that the repetitive flipping activity is a response to being reared in a captive, laboratory environment, rather than simply being housed for a prolonged time in captivity; whereas the time spent gnawing may be a more inflexible component of the activity cycle. Selection against the combined Flipping + Gnawing score resulted in rapid response in Flipping, with the behavior extinguished almost completely by G6 in the DOC stocks (Figure 3).

The behavioral traits and each measured component of reproduction showed significant changes across generations in at least some populations, and often the direction of the change was not the same in all protocols and replicates (Table 2). To quantify the changes across generations, at each level of partitioning (overall, by Protocol, and by Protocol-Replicate), regressions against generation were forced to have a common intercept; the intercept from the Overall model is given in Table 2. Regressions for Flipping and Gnawing included sex as a covariate, and intercepts are the predicted values at G0 for males. Regressions for Litter Size, Litter Survival, and Weaning Mass included litter number as a categorical predictor variable, and intercepts are the predicted values for the 1^st^ litter.

**Table 2:** Effects of Generation on measures of activity and reproductive performance, for all subjects (Overall model), for each Protocol (across both Replicates), and for each Replicate of each Protocol.

**Population**	**Flipping^b^**	**Flipping (G0-G9)^c^**	**Gnawing^b^**	**Pr[Breed]^a^**	**Delay^b^**	**Litter Size^b^**	**Litter Survival^a^**	**Weaning Mass^b^**	**Repro. Success^b^**
Intercept	119.2	80.71	79.1	0.430	38.28	4.22	0.882	10.82	40.32
Overall	4.98***	10.47***	-0.86***	0.027***	-0.38***	-0.01*	-0.006***	-0.05***	1.06***
RAN	7.95***	18.53***	-1.18***	0.029***	-0.42***	-0.01	-0.006***	-0.06***	1.09***
RAN1	2.33***	16.93***	0.71**	0.021***	-0.29***	-0.04***	-0.003	-0.09***	0.14
RAN2	11.59***	20.90***	-2.12***	0.032***	-0.45***	0.02***	-0.009***	-0.05***	1.61***
MK	3.84***	10.36***	-0.47**	0.037***	-0.40***	-0.01***	-0.006***	-0.03***	1.74***
MK1	3.13***	14.08***	-0.72***	0.035***	-0.35***	0.00	-0.006***	-0.08***	1.40*
MK2	3.76***	7.84***	0.41	0.034***	-0.36***	-0.03***	-0.006***	0.01	1.65***
DOC	-10.42***	-9.01***	-2.61***	0.010**	-0.06	-0.03***	-0.006***	-0.13***	-0.77*
DOC 1	-10.82***	-9.96***	-2.56***	0.010**	-0.05	-0.03***	-0.006**	-0.13***	-0.71**
DOC 2	-12.37***	-2.96	3.74**	-0.038***	1.07*	-0.05	-0.001	-0.25***	-4.73**

Asterisks indicate statistical significance: *P < 0.05; **P < 0.01; ***P < 0.001.

^a^ For dichotomous variables (Pr[Breed] and Litter Survival) effects are expressed as derivatives of the change from logistic regressions, with the effect being the difference between the predicted value for G1 and G0.

^b^ For continuous variables, effects are the slopes from linear regressions.

^c^ For Flipping behavior, regressions are reported across both the full 18 generations and for the first 9 generations, during which the DOC 2 population persisted and over which most of the change in Flipping occurred in all populations.

**Figure 4:**
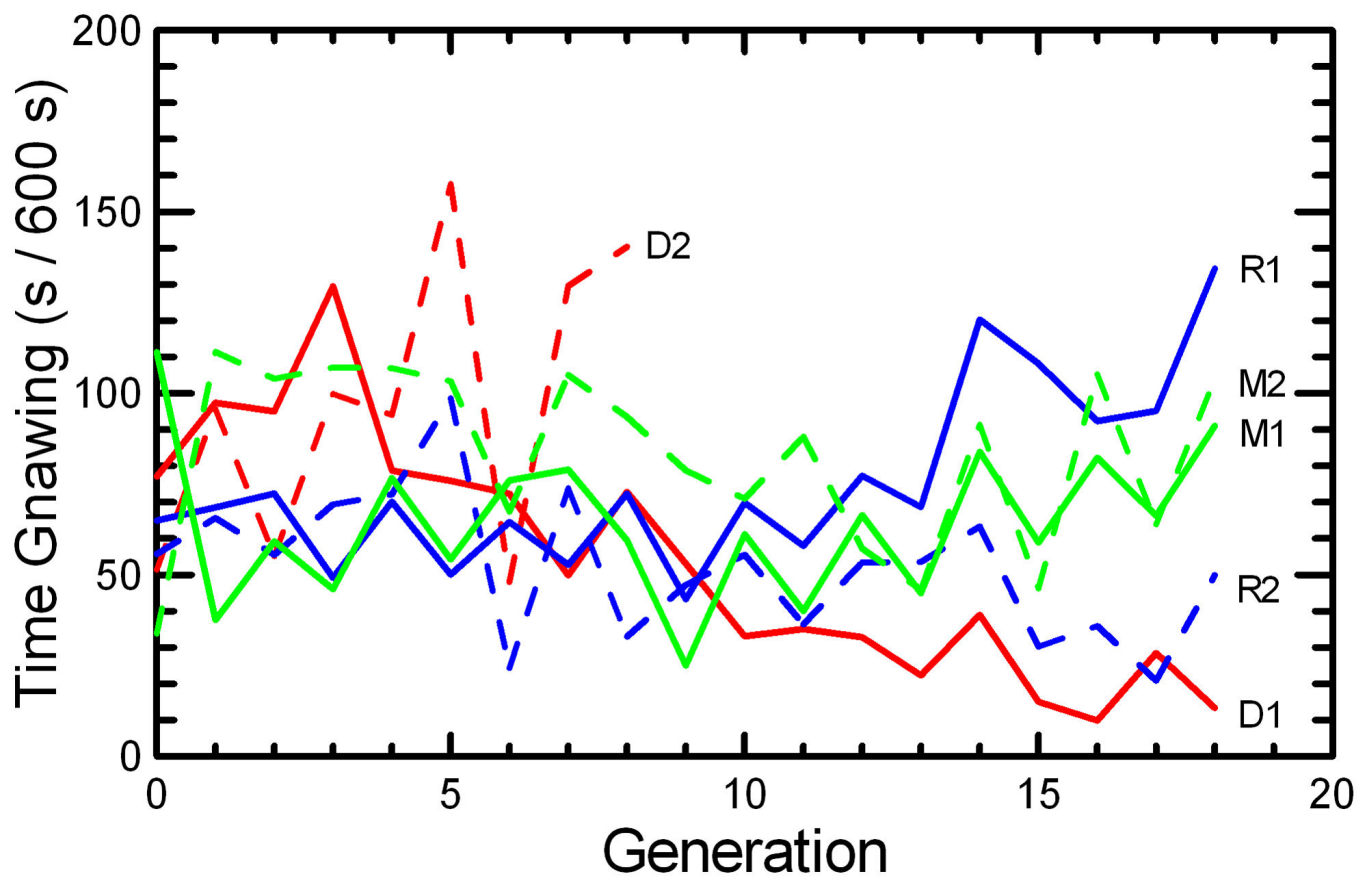
Mean time spent in stereotypic Flipping behavior. The mean number of seconds per 600 s observation period that mice spent in Gnawing behavior is given for the two replicates of RAN (blue), MK (green), and DOC (red) experimental populations.

The linear regressions in Table 2 over the full 18 generations obscure the fact that the DOC 1 had little scope for further reduction after G8, Flipping appeared to plateau in the MK populations, and it decreased after G9 in RAN1 (Figure 3). Therefore, the regressions for Flipping were repeated on just the first 9 generations of captive breeding, during which the DOC 2 population persisted. Flipping increased significantly and consistently in the unselected RAN and MK stocks, resulting in about a two-fold to three-fold increase by G8. Little further change occurred in the MK populations in subsequent generations, while the two RAN replicates diverged. Changes in Gnawing were inconsistent across Replicates, with some divergence among populations and a decrease in DOC 1 after the extinguishing of Flipping left selection only on the Gnawing (Figure 4).

Although large differences in the patterns of response to selection were observed (Figures 3 and 4), the differences among Protocols in the change over the full 18 generations were not significantly greater than the very large variation among Replicates (Table 3). When analyzed only over the first 9 generations, the differences in the trends in Flipping among Protocols were highly significant when tested against variation between Replicates. Gnawing showed significant variation among Replicates, but no consistent differences between Protocols, throughout the breeding study.

**Table 3:** Statistical tests (mean squares and appropriate F-tests from generalized linear models) of effects of sex, breeding protocol, replicate, and generation on stereotypic behavioral traits of Flipping and Gnawing.

	**Flipping (G0-G18)**		**Flipping (G0-G9)^a^**		**Gnawing (G0-G18)**	
**Effect^b^**	**MS**	**F-test**	**MS**	**F-test**	**MS**	**F-test**
Sex	233242	6.20*	1210	0.03	103238	7.59**
Protocol	10777700	2.84	4989600	5.43	97072	0.23
Replicate{Protocol}	3794350	100.9***	919736	26.5***	415775	30.6***
Generation	1943230	51.7***	2273980	65.4***	131313	9.66**
G* Protocol	1417520	0.74	2436870	35.9***	433237	1.38
G* Replicate{Protocol}	1915000	50.9***	67890	1.95	313709	23.1***
Residual (df)	37607	(9922)	34756	(4337)	13595	(9922)

Asterisks indicate statistical significance: *P < 0.05; **P < 0.01; ***P < 0.001.

^a^ Analysis of Flipping scores were repeated for just the first 9 generations, during which most changes in Flipping occurred.

^b^ The top four effects were include in all models, the interaction effects were tested in further models, and the Residual MS is from the last, most complete model.

### Effects of generations of laboratory breeding on reproduction

**Figure 5:**
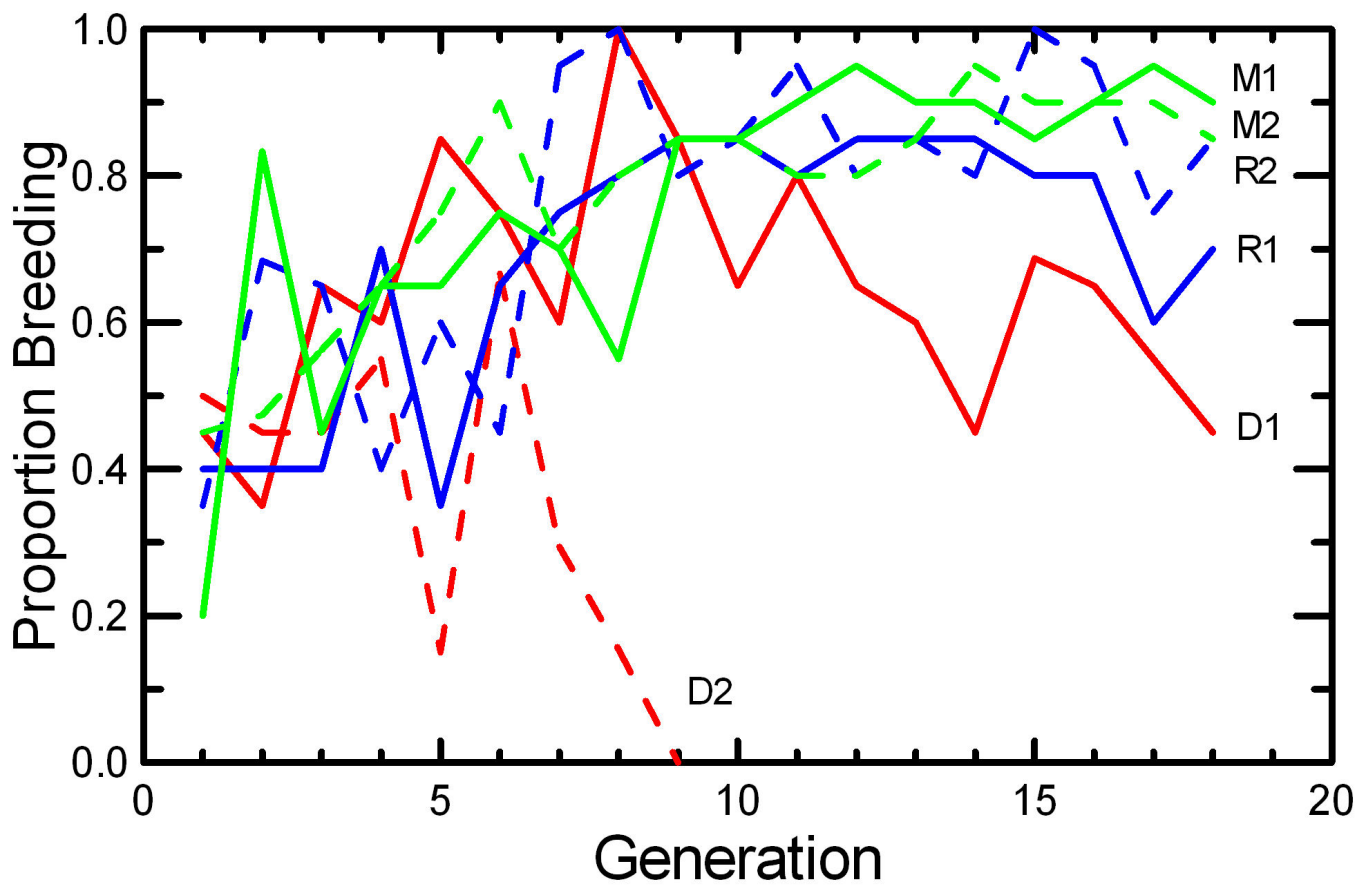
Probability that a pair will breed. The proportion of pairs that produced at least one litter conceived within 70 days is given for the two replicates of RAN (blue), MK (green), and DOC (red) experimental populations.

The probability that a pair produces a litter, Pr[breed], showed highly significant increase across generations, a highly significant variation in this overall trend among protocols, and significant variation in the effect of Generation among replicates within protocols (G*Replicate{Protocol}) due to a marked decrease in DOC 2 (Tables 2 & 4). In the RAN and MK Protocols, Pr[breed] increased from less than 50% at G1 to more than 80% at G9 (Figure 5). After G9, there was little scope for further increases in the probability of breeding in the RAN and MK populations. In the DOC protocol, Pr[breed] initially increased and then decreased in DOC 1, and decreased to 0 by G9 in DOC 2. Across measures of reproduction, the overall effect of the DOC protocol (Table 2) was dominated by DOC 1 due to the absence of DOC 2 in the latter generations.

**Table 4:** Statistical tests of effects of sex, breeding protocol, replicate, and generation on dichotomous measures of reproductive performance, from logistic regression models.

	**Pr[Breed]**		**Litter Survival**	
**Effect^a^**	**-LL**	**G-test^b^**	**-LL**	**G-test^b^**
Protocol	1134.38	53.6***	940.05	2.28
Replicate{Protocol}	1118.24	32.3***	932.97	14.2**
Generation	1077.97	80.5***	923.97	18.0***
G* Protocol	1055.84	44.3***	923.54	0.86
G* Replicate{Protocol}	1050.00	11.7*	923.53	0.01

Asterisks indicate statistical significance: *P < 0.05; **P < 0.01; ***P < 0.001.

^a^ Each model includes all effects in the models above it.

^b^ G-test values are twice the change in the log-likelihood (LL) of the model when the effect was added to the model.

**Figure 6:**
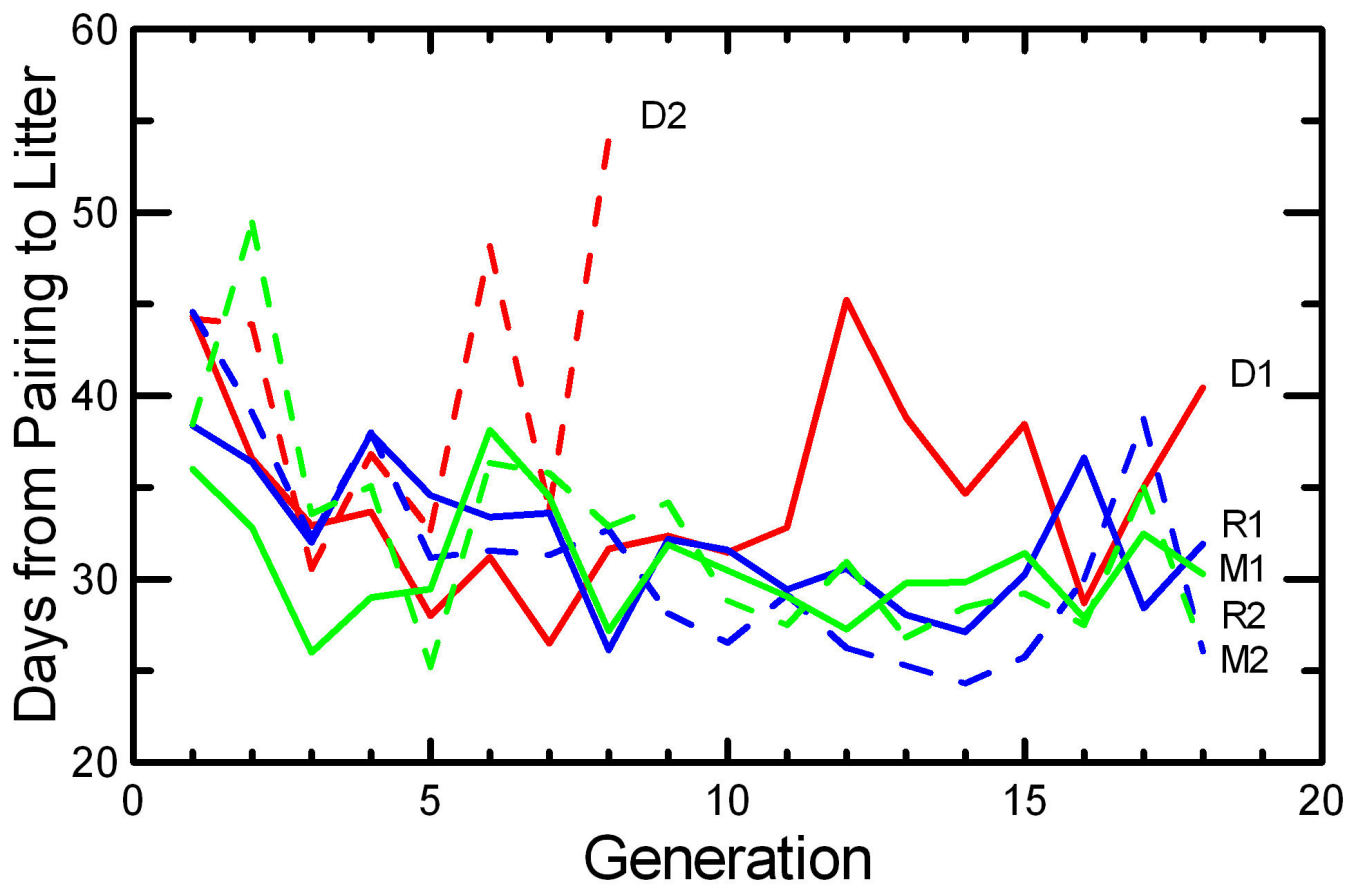
Delay between pairing and litter birth. The mean time (d) between pairing and the birth of a litter for those pairs that produced at least one litter is given for the two replicates of RAN (blue), MK (green), and DOC (red) experimental populations. Note that the y-axis starts at 20 d, a few days shorter than the typical gestation period.

The lag time between pairing and the birth of the first litter (Delay) decreased across the generations of laboratory breeding (Table 5), with a reduction from about 40 days to about 30 d occurring in the first 5 generations (Figure 6). Gestation is about 23 d, so the average delay between pairing and conception decreased from about 17 d to 7 d. Breeding was significantly more rapid in both the RAN and MK protocols (Table 2), but the trend was weak and non-significant in the DOC protocol (and reversed in DOC 2).

**Table 5:** Statistical tests of effects of breeding protocol, replicate, and generation on continuous measures of reproductive performance of pairs, based on ANOVAs from generalized linear models.

	**Delay**		**Litter Size**		**Wean Mass**		**RS**	
**Effect^a^**	**MS**	**F-test**	**MS**	**F-test**	**MS**	**F-test**	**MS**	**F-test**
Protocol	1521	4.38	2.65	0.05	105.4	1.83	69599	5.64
Replicate{Prot}	348	2.12	49.2	30.4***	57.8	17.6***	12336	5.89***
Generation	2663	16.2***	9.43	5.83*	185.0	56.3***	30996	14.8***
G* Protocol	804	2.82	4.31	0.26	7.7	0.12	40182	3.38
G* Replicate{Prot}	285	1.73	16.9	10.4***	64.0	19.5***	11878	5.67***
Residual (df)	164.2	(1337)	1.617	(2463)	3.289	(2216)	2096	(1910)

Asterisks indicate statistical significance: *P < 0.05; ***P < 0.001.

^a^ The top three effects were include in all models, the interaction effects were tested in further models, and the Residual MS is from the last, most complete model.

**Figure 7:**
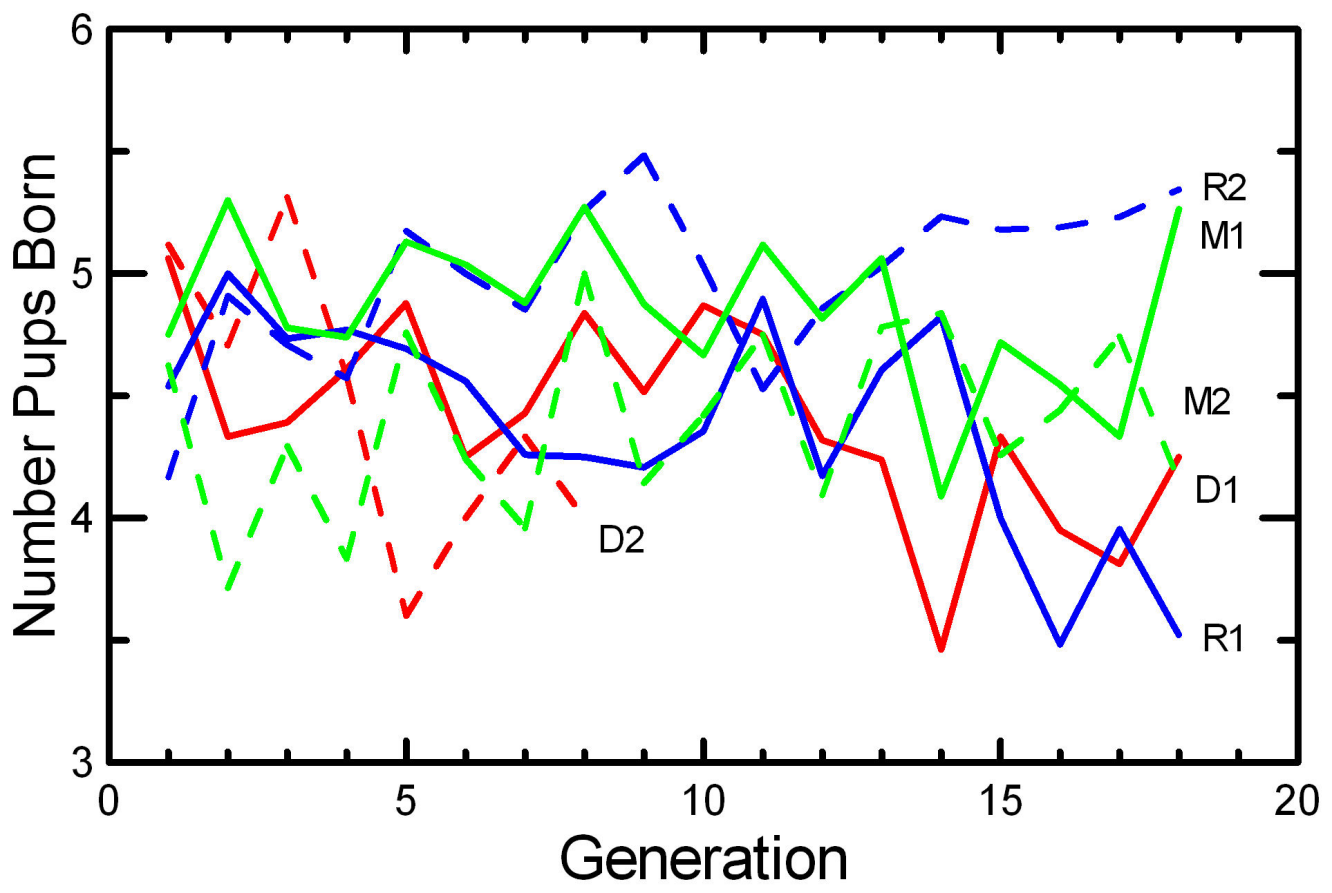
Mean litter size. The mean number of pups born in first and second litters is given for the two replicates of RAN (blue), MK (green), and DOC (red) experimental populations.

**Figure 8:**
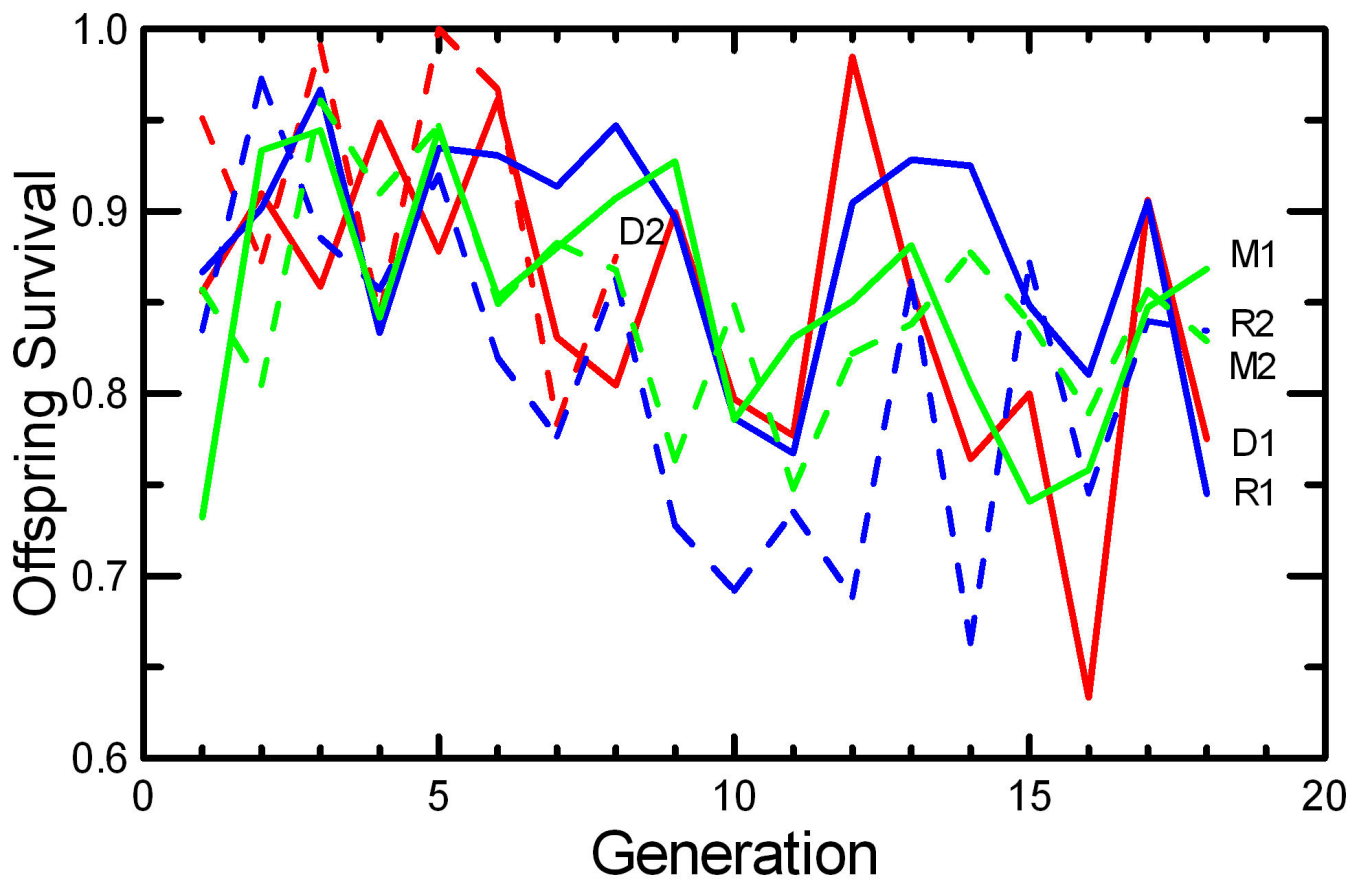
Litter survival to weaning at 20 days. The mean survival of litters (with each litter scored as survival = 1 if more than 50% of pups survive, and survival = 0 otherwise) is given for the two replicates of RAN (blue), MK (green), and DOC (red) experimental populations.

**Figure 9:**
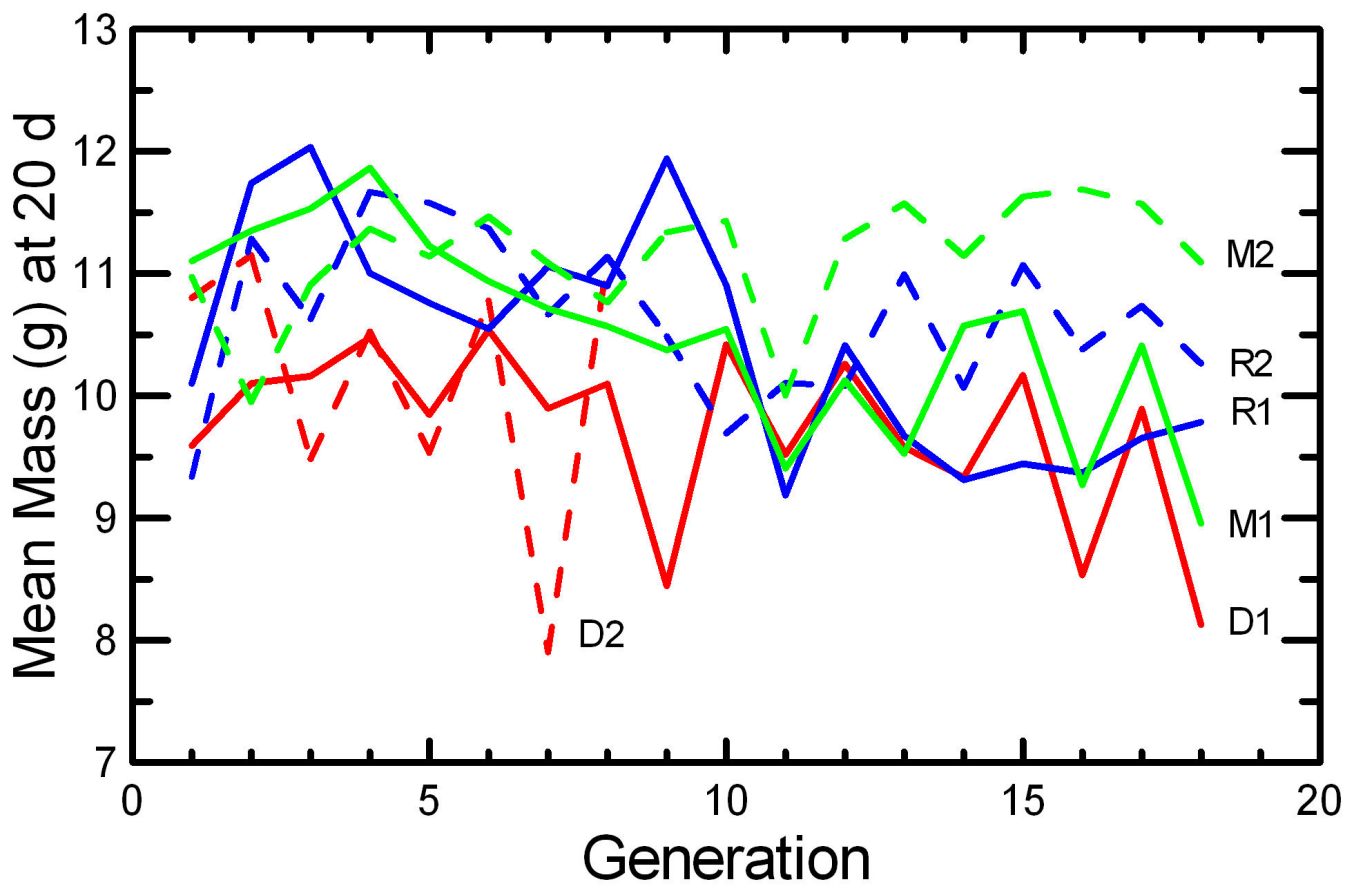
Weaning Mass. The mean mass (g) of pups weaned for those pairs that produced at least one litter is given for the two replicates of RAN (blue), MK (green), and DOC (red) experimental populations.

**Figure 10:**
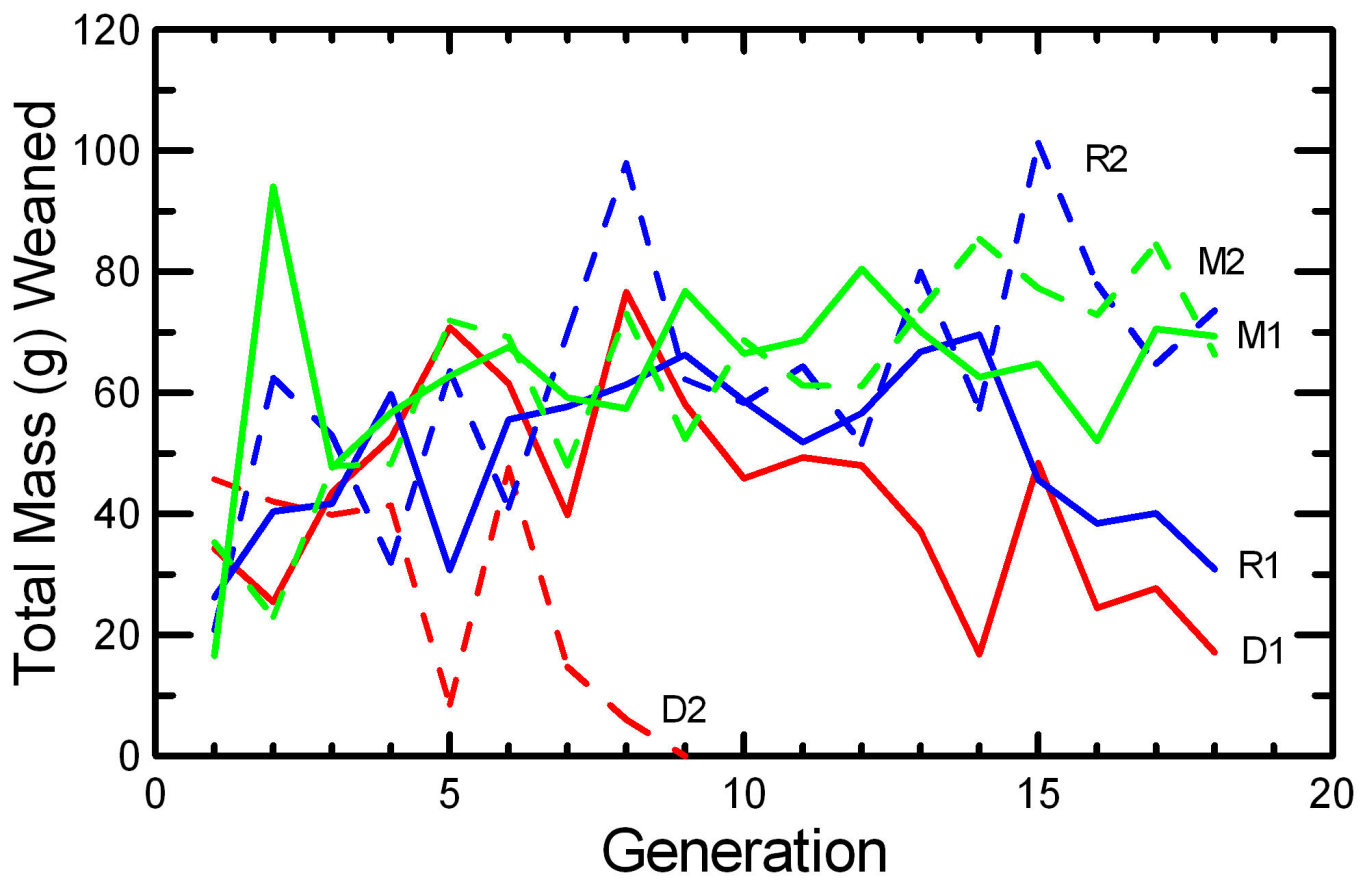
Overall reproductive success. The mean total mass (g) of progeny weaned per pair in the 0, 1, or 2 litters produced is given for the two replicates of RAN (blue), MK (green), and DOC (red) experimental populations.

Litter Size decreased in DOC populations and in one replicate each of MK and RAN (Figure 7; Table 2), resulting in significant effects of Generation, Replicate, and Generation*Replicate (Table 5). There was a highly significant decrease in Litter Survival across generations (Figure 8), consistent in both replicates of all three protocols (Table 4). The trend was not significant in every population (Table 2), but the trends were similar and there were no significant interactions of Generation with Protocol or Replicate. Similarly, the mean Weaning Mass of pups showed a significant decrease across Generations overall, in all three protocols, and in all replicates except MK2 (Tables 2 and 4; Figure 9). The total mass of pups weaned by a pair (RS) showed strong increases across Generations in RAN2 and both replicates of MK, but decreased in both DOC replicates (Figure 10; Table 2), resulting in significant effect of Generation, Replicate, and Generation*Replicate (Table 5). RS increased by about 70% over the 18 generations of laboratory breeding in both MK replicates and RAN2 (Table 2). Overall reproductive success decreased by 29% in DOC 1, and DOC 2 had complete reproductive failure by G9.

### Impacts of behavior on reproduction

Selection against Flipping and Gnawing in the DOC protocol resulted in rapid reduction in Flipping. There was only a smaller reduction in Gnawing that occurred after G8 in DOC 1, when almost no Flipping persisted so that all the selection was then focused on the Gnawing score (Figure 4). The non-selected RAN and MK stocks showed rapid increase in Flipping but inconsistent changes in Gnawing (Table 2). Thus, in contrast to an assumption made when the study was designed that both Flipping and Gnawing were similarly stereotypic and likely maladaptive responses to captivity, natural selection in the captive environment must have favored the flipping behavior, while the gnawing behavior appeared resistant to change due either to artificial (DOC) or natural selection. To test what components of fitness were associated with Flipping, regressions (either linear or logistic, as appropriate) of measures of reproductive performance were repeated with Flipping score of the dam and of the sire added to Protocol, Replicate, Generation, and Generation*Replicate-Protocol as predictor variables. Every component of reproductive success was affected by the Flipping score of the dam, with regression coefficients expressed as changes per 60 s (10% of the observation time) of Dam Flipping: Pr[breed] b = 0.055(P < 0.001); Delay b = -0.58(P < 0.001); Litter Size b = 0.028(P < 0.001); Litter Survival b = 0.006(P < 0.01); Weaning Mass b = 0.187(P < 0.001); and RS b = 4.57(P <0.001). These strong positive associations of Dam Flipping with reproduction would have been selective forces that led to the rapid increase in Flipping in RAN and MK populations, as well as to the reproductive decline in the DOC populations selected for low Flipping + Gnawing. Sire Flipping affected only Litter Survival b = -0.010(P < 0.0001), with the effect being the reverse of that observed for Dam Flipping. Similar analyses of Gnawing showed no significant effects of dam and sire Gnawing scores on reproductive performance.

### Effects of inter-crossing replicates

In generations 19 and 20, crosses were made between the two replicates of RAN and of MK to reverse the accumulated inbreeding and to indicate if the changes that occurred through generations of captive breeding had additive effects. By G18 all mice had ancestry from all 20 progenitors. However, the lack of more recent common ancestry between mice in different populations kept the between-population kinships low and meant that most of the inbreeding accumulated within populations could be immediately reversed by inter-replicate crosses. Table 6 shows the sample sizes, mean inbreeding, and Flipping and Gnawing scores for the first generation in which populations were distinct (G1), the last two generations of mice from isolated replicates (RAN1, RAN2, MK1, and MK2), the inter-replicate crosses (designated RANx1 and MKx1, conducted over two generations), and second-generation crosses (RANx2, MKx2). The low inbreeding in the RANx1 and MKx1 crosses reflected the common ancestry from G0, and second generation crosses had inbreeding levels intermediate between the initial crosses and the parental replicate stocks.

**Table 6:** Mean inbreeding and activity scores (SE) for mice in the starting population (G1), final two generations of each experimental population (RAN1, RAN2, MK1, and MK2), and two generations of intercrosses between replicates of each protocol (RANx1, RANx2, MKx1, and MKx2).

**Population-Generation**	**N**	**Inbreeding**	**Flipping**	**Gnawing**
G1	380	0.00	78.4 (8.7)	62.6 (6.6)
RAN1-G19/20	60	0.28	186.2 (28.8)	104.7 (21.9)
RAN2-G19/20	69	0.27	345.9 (23.2)	10.9 (5.2)
RANx1-G19/20	263	0.02	222.8 (14.1)	61.7 (7.6)
RANx2-G20	147	0.15	246.0 (18.0)	65.5 (11.6)
MK1-G19/20	50	0.22	175.6 (32.0)	53.8 (10.2)
MK2-G19/20	76	0.19	160.6 (21.7)	70.3 (12.8)
MKx1-G19/20	287	0.03	127.9 (12.2)	79.5 (7.5)
MKx2-G20	145	0.12	111.3 (16.9)	75.2 (11.0)

The inter-replicate crosses had Flipping scores intermediate between the baseline generation and the last generations of the parental populations (Table 6). This indicates that the increases in Flipping across generations were not due to evolution of genes with solely additive effects, but instead the changes in behavior were due in part to parallel evolution of either epistatic interactions among loci or of recessive alleles that would then be partly masked in the crosses. The lack of reversion of second generation crosses (with partly restored inbreeding) to the Flipping levels of the G19/20 parental replicates indicates that the increase in Flipping in RAN and MK populations was not an effect of inbreeding itself. The Gnawing scores in inter-replicate crosses were not significantly different than the mean of the respective parental replicates, nor significantly different than the G1 baseline. The intercrosses appeared to reverse the random divergence among replicates in Gnawing that had accumulated over the generations.

Table 7 provides the sample size (number of pairs) and measures of reproductive performance for the first and last generations of replicates and the inter-crosses. Across the two protocols and 6 measures of reproductive performance, only for Weaning Mass in MK was there a significant difference between the first (MKx1) and second generation (MKx2) of the crosses. Only for Weaning Mass and RS were there significant differences between the intercrosses and their parental replicate populations; in both cases the intercrosses outperformed the mean of the parental replicates. For the three components of reproduction that showed highly significant improvements across 18 generations in RAN and MK populations (Pr[Breed], Delay, and RS), the intercrosses maintained the improved performance of the prior generations and continued to outperform the G1 baseline (P < 0.001 for all three components). In the three measures that showed significant declines across 18 generations (Litter Size, Litter Survival, and Weaning Mass), the mean of the intercrosses was higher than the mean of the G18/G19 replicates and higher than the G1 baseline, suggesting that the reversal of inbreeding in the intercrosses reversed the small declines that had occurred in these components of reproduction. However, large fluctuations among generations and variation among replicates makes identification of patterns difficult, and the improvement in the intercrosses over the prior generation replicates (P < 0.01) and over the baseline (P < 0.05) was significant only for Weaning Mass.

**Table 7:** Means (SE) of measures of reproductive performance for pairs creating the starting population (G1), final two generations of each experimental population (RAN1, RAN2, MK1, and MK2), and two generations of intercrosses between replicates of each protocol (RANx1, RANx2, MKx1, and MKx2).

**Population-Generation**	**N**	**Pr[Breed]**	**Delay**	**Litter Size**	**Litter Survival**	**Weaning Mass**	**Repro. Success**
G1	120	0.39 (0.04)	41.5 (2.6)	4.25 (0.13)	0.81 (0.03)	10.2 (0.2)	29.8 (3.9)
RAN1-G18/19	30	0.73 (0.08)	30.0 (2.4)	3.28 (0.25)	0.79 (0.05)	9.6 (0.4)	39.2 (6.7)
RAN2-G18/19	30	0.84 (0.07)	28.5 (1.5)	4.52 (0.17)	0.76 (0.05)	10.3 (0.2)	71.2 (9.2)
RANx1-G19/20	40	0.90 (0.05)	30.9 (2.4)	4.23 (0.16)	0.79 (0.04)	10.5 (0.3)	70.7 (7.9)
RANx2-G20	20	0.80 (0.09)	27.1 (1.1)	4.41 (0.25)	0.90 (0.03)	10.9 (0.3)	80.9 (10.6)
MK1-G18/19	30	0.80 (0.07)	33.8 (3.6)	4.70 (0.22)	0.84 (0.04)	9.1 (0.3)	63.0 (8.1)
MK2-G18/19	30	0.90 (0.06)	31.1 (2.8)	3.67 (0.15)	0.81 (0.04)	11.0 (0.2)	72.6 (7.4)
MKx1-G19/20	40	0.85 (0.06)	30.7 (1.9)	4.06 (0.15)	0.88 (0.03)	11.4 (0.2)	82.2 (7.7)
MKx2-G20	20	0.85 (0.08)	29.0 (1.9)	4.43 (0.19)	0.83 (0.05)	9.8 (0.4)	71.4 (10.2)

## Discussion

We monitored changes in behavior and reproductive patterns over 18 generations of captive propagation of *Peromyscus* *leucopus* mice, starting from a common genetic stock created from 20 mice captured in a natural habitat. The mice were propagated under three breeding protocols: a strategy that maximally maintains gene diversity; artificial selection that represented the selection that might be pursued deliberately or unintentionally in attempts to adapt wildlife to captivity; and random breeding. Each breeding protocol was practiced on two replicate populations to allow assessment of the roles of drift vs. artificial selection vs. natural selection in causing changes through the generations. The use of a common founder stock precluded the possibility that founder effects (differences between lines arising either from random sampling of founders to create experimental populations or from establishment of populations from distinct and often undocumented wild populations) were the cause of observed differences – a potential problem that exists in many studies of the effects of captivity [36,37]. Housing, handling, breeding, and data collection protocols were carefully controlled, and possible confounding factors of sex and litter number (parity) were treated as covariates in analyses to control statistically for those effects.

The breeding protocols mimicked the protocols of many captive breeding programs for wildlife species. The MK protocol is the standard method of genetic management for breeding programs regulated by the regional zoo associations [38]. The random breeding protocol is perhaps closest to the “management” of most wildlife species in captivity, in which there is neither rigorous management for gene diversity nor active selection for specific traits. The “Docility” protocol represents the selection that would occur either because caretakers favor animals that are easier to handle [39] or because artificial selection is practiced against animals that show the stereotypic behaviors sometimes seen in captive animals and presumed to indicate that those animals are not well adapted to the captive environment. Indications of stress and responses that are maladaptive are not easy to predict or measure, but repetitive locomotion is often interpreted to indicate harmful stress in captivity [40]. Stereotypies in captive animals are often considered to be synonymous with poor welfare, although studies of individual differences within populations under common environmental treatments [41] found that more often than not the individual animals with highest rates of stereotypic behavior had the best scores on other measures of welfare.

Although we could have used selection on any arbitrary trait to explore the effect of artificial selection on populations adapting to a novel captive environment, the Flipping and Gnawing behaviors were chosen for study because we surmised (as have others [30]) that these consistent, repetitive behaviors were responses to the stress of being confined in a small cage. Such behaviors might be associated with poor physical or psychological condition of the animals, and could represent frustrated attempts to escape by mice poorly adjusted to the captive environment [42]. Even if not responses to harmful stress, stereotypies could reduce fitness simply as a result of animals spending more time in repetitive behaviors putting fewer resources into reproduction. McDougall et al. [39] suggested that in general highly active animals are less well adapted to captivity, and they noted the docility of laboratory strains of rodents as evidence. However, during the development of lab strains (most of which were derived from pet trade stocks), lab mice and rats have almost certainly been strongly selected for ease of handling whether or not this was adaptive for the animals.

In striking contrast to expectations, we found that one of the presumed stereotypic behaviors was positively associated with reproductive fitness within each population. Selection on the docility traits caused marked reduction in reproduction, and populations not under artificial selection to reduce these behaviors responded with large increases in the time spent Flipping. Response to artificial selection was rapid, with almost complete extinguishing of Flipping within 5 generations. However, the response was primarily in only one of the two traits on which selection was imposed, with Gnawing showing a slow decrease in DOC 1 starting only in generation 8, by which time the selection was being imposed almost solely on Gnawing. Moreover, the reduction in Flipping was achieved in spite of apparently strong natural selection favoring that behavior. Indeed, the positive change in Flipping in the RAN and MK populations over the first 8 generations was almost as rapid as the negative response to artificial selection against the behavior in DOC, resulting in mice in RAN and MK populations that, on average, spent a third to half of the active time engaged in the flipping behavior. We do not know if the positive association of flipping with fitness is due to a benefit of the flipping or if instead animals with high fitness otherwise might engage in more flipping (perhaps due to just higher activity levels). Würbel [30] reviewed various hypotheses regarding the motivations of such stereotypies in rodents, including a stress-coping mechanism, thwarted natural behavior, attempts to escape or seek shelter, stress-induced behavioral sensitization, and pathological brain dysfunction. While data do not exist to discriminate among all of the hypotheses, Würbel concluded that it was “rather unlikely” that the “stereotypies might be an effective means of coping with environmental restrictions, with stereotyping animals being better off”. Our study, however, indicates that *Peromyscus* with higher rates of stereotypic flipping do have higher fitness. It is notable that our wild-caught mice almost never engaged in flipping in the cages; the causes and effects of this captive-adaptive behavior in *Peromyscus* *leucopus* merit further study. Note that by describing flipping as captive-adaptive, we do not mean that the flipping behavior itself necessarily benefits the mice (although it might). The genes that lead to more flipping in captivity might have positive fitness effects due to pleiotropic effects on other traits.

Rapid evolutionary response to the captive environment was also seen in some aspects of reproduction. Malo et al. [43] have previously reported that as of generation 10, the DOC 1 mice had a higher rate of sperm abnormalities and that these correlated with lower fertility. In the non-selected lines, across the 18 generations of captive breeding, there was a much shorter delay between pairing and conception and a much greater proportion of pairs that produced litters (Tables 2, 4, and 5). In an early study of 25 generations of captive breeding of wild Norway rats (*Rattus norvegicus*), King [18] found a similar acceleration of breeding, with reduced sterility, earlier age of breeding, more litters, and improved parental care, although she had actively selected for higher fecundity and faster growth rate. In a study of a laboratory colony of *Peromyscus* *maniculatus* that was selected for rapid reproduction, Millar and Threadgill [44] reported inconsistent changes in reproductive performance across 11 generations, and they did not interpret the changes as having been responses to selection. They did not report the rate of inbreeding, and it may have been high enough to cause inbreeding depression in later generations. No replicate populations were used to confirm that their results were not due to random drift.

In our laboratory breeding procedures, all pairs received the same number of days to breed, the time permitted for breeding (70 days) was much longer than the mean time to conception (about 14 days in the early generations), each pair was allowed to produce only two litters, and the subsequent generation was not paired until all progeny had become fully adult. These methods would have precluded the selection for rapid breeding that would result from a scheme in which animals were paired for breeding as they became available, or had only limited time to breed, or were allowed to produce as many litters as possible within the time paired. However, although there would not have been direct selection for rapid breeding in our study, there would have been very strong selection against choosiness by either sex during mating. *Peromyscus* *polionotus* mice have been shown to have mate preferences that are influenced by kinship and impact reproductive success [45]. We provided each mouse with a single possible partner with which to mate. Any failure to mate due to behavioral or physiological unacceptability of the partner provided would constitute intense selection against such mate choice behavior and against any physiological incompatibility mechanisms. Further studies would be needed to test if mate choice had been diminished in the RAN and MK mice, and if this was the mechanism by which delay from pairing to conception became much shorter and the proportion of pairs breeding rose from about 40% to more than 80%.

Given the rapid evolution of reproductive patterns over just the first 5-10 generations in captivity, any studies that use long-established laboratory strains to study reproductive behavior should be aware that the patterns observed might be responses to the highly altered selection on reproductive patterns of the captive environment, and possibly not reflective of adaptations evolved in natural populations of the species. Millar and Threadgill [44] made the same caution and recommended that the number of generations in captivity be considered as a covariate in any studies of reproduction and development.

The adaptation of the reproductive pattern to captivity did not occur in the docility-selected populations. Mice that do not flip at night also are more reluctant to breed, relative to the RAN and MK mice that evolved toward high and rapid pairing success. Further behavioral studies are needed to explore what other behavior patterns are associated with the flipping behavior. McPhee [36] studied timidity (assessed by exploratory behavior, activity level, and enclosure use) in *Peromyscus* *polionotus* populations that had been in captivity for 0, 2, 14, and 35 generations. She found no evidence for changes in mean activity and timidity, and no behavioral change in the first 14 generations, and but did observe changes in the variability among mice at 35 generations. Belyaev [17] conducted a long-term study of selection for tameness in silver foxes from fur farms. He found that a number of morphological and reproductive traits, including increased fertility, changed along with the selected trait (lack of fearfulness of humans). Although the foxes evolved dog-like traits and greater variation that might be characteristic of domestication (reproductive adaptation to captivity and lack of aggression toward humans), the apparently subjective means of selecting breeders leaves uncertain if the observed changes were all due to the selection on tameness or could also have been due to inadvertent selection for other traits.

One purpose of the MK breeding strategy is to minimize natural selection for captive adaptive traits [25], by keeping the future genetic contribution of each founder lineage as constant as possible [22,38]. Across the traits that showed directional change indicating adaptation to captivity (Flipping, Br[Breed], and Delay), there was not a clear trend for the MK populations to show any less adaptation than the RAN populations; RAN2 usually performed comparably with MK1 and MK2, while RAN1 performed more poorly, especially in the last few generations. For a number of the traits measured, the MK populations showed less divergence between replicates and more consistency across generations than did the RAN populations. Thus, over 18 generations of captive breeding, there was evidence that the MK strategy reduced inbreeding and reduced random drift and divergence of populations, but no evidence that it effectively countered selection for captive-adapted traits. Similarly, breeding strategies to minimize kinship did not slow adaptation to captivity in *Drosophila* relative to randomly bred controls [26].

In the novel captive environment, if natural selection is free to act on the population, we would expect evolution toward greater fecundity and higher offspring survival, as the captive environment and protocols largely remove some constraints such as food limitations and trade-offs of lower current reproduction for greater adult survival and future reproduction. However, we saw no increase in the number of pups born per litter, survival of litters, or growth rate of pups, and all three of these measures of reproductive output decreased significantly over the 18 generations. This suggests that there was no additive genetic scope for increase in fecundity. The observed declines in reproductive output could have been due to inbreeding depression in the closed populations. Studies on other *Peromyscus* species have reported no change in litter size through generations of captive breeding [46–48].

Within-population, within-generation correlations showed that Flipping behavior had a strongly positive association with almost all components of reproductive fitness in captivity. After about G6, the lack of productivity in DOC 2 may have led to a feedback in which inbreeding depression exacerbated the negative effects of the artificial selection. Thus, after the DOC 2 population exceeded a mean inbreeding level of about F = 0.15, poor reproduction sealed the fate of that population, although other populations in which inbreeding accumulated more slowly reached F = 0.20 to F = 0.30 by G18. In later generations of this study, the DOC 1 replicate showed a trend toward lower performance in the same measures of reproduction that contributed most to the earlier collapse of DOC 2. RAN1 also showed declines in weaning mass and RS in the last few generations.

Any small, closed population will inevitably accumulate inbreeding. Therefore, it would be easy to confuse adaptation to the novel laboratory environment with an apparently positive effect of inbreeding. For example, the large increase in the probability of breeding and the shorter delay from pairing to conception across 18 generations in this study (Figures 5 and 6; Table 2) could have been interpreted as a positive benefit of increased inbreeding or kinship among mice if we had simply reported correlations of fitness and inbreeding pooled across all generations. However, other studies of this species [49] and congeneric species [50] found that when deliberately inbred pairs were contrasted with non-inbred pairings within each generation, inbreeding caused significant reductions in almost all components of reproductive success. At the end of the present study, inbreeding was reversed with inter-crosses between replicates. The components of reproduction that had improved across the generations (Pr[Breed], Delay, and RS) maintained their levels after the inter-crossing, demonstrating that these changes across generations were due to genetic adaption to captivity rather than to any benefit of inbreeding or close kinship between paired mice. Other components of reproduction (Litter Size, Litter Survival, and Weaning Mass) decreased across generations (Figures 7, 8, and 9; Table 2), and the reductions were comparable to what would be predicted based on the magnitude of inbreeding depression in these traits reported by Brewer et al. [49] for this species. The likelihood that the reductions in these components of reproduction were due to inbreeding is supported by the observation that the changes were reversed in the inter-crosses between replicates (Table 7). Given the rapid changes in both behaviors and reproductive patterns that we observed across generations in captivity, we would urge caution in interpreting trends with inbreeding from studies of captive populations unless any effects of adaptations to captivity can be controlled by measuring subjects at different levels of inbreeding that are the same number of generations removed from the wild.

Overall, the changes in behavior and some aspects of reproduction were significant and large across generations, and the populations responded strongly to natural selection favoring flipping and to artificial selection against the behavior. Almost every trait showed significant change across generations, when pooled across Protocols and Replicates, although often the magnitude and even direction of change was different in the DOC selected populations relative to the MK and RAN populations. Differences among populations in the responses could have been due in part to random genetic drift, rather than differences in the breeding protocols. Only for the reproductive trait most strongly responding to captivity, Pr[Breed], was the difference between Protocols in the trend across generations significant when tested against Generation * Replicate{Protocol}.

The inter-replicate crosses demonstrated that changes in reproduction that we interpret as responses to natural selection (in Pr[Breed] and Delay) were preserved when replicate populations were crossed for two generations, suggesting that these changes were due mostly to additive effects that were not disrupted when previously isolated populations were crossed. The time spent in flipping behavior regressed partially back toward baseline means, indicating that the changes in flipping were not caused by evolutionary shifts in the same set of alleles in the different replicate populations and that the effects were not entirely additive.

Various authors have suggested that artificial selection for traits that are expected to be associated with high fitness in captivity could sometimes be practiced to enhance success of breeding programs (e.g., [8,39]), although the authors often include cautions about the risks of such an approach. However, we found that we had naïve expectations about what behaviors are adaptive responses to captivity versus maladapted manifestations of harmful stress. Artificial selection that we expected would accelerate adaptation to captivity by eliminating apparently stereotypic repetitive behaviors instead caused reproductive failure due to elimination of a behavior that was actually a highly adaptive response to captivity (through unknown mechanisms). In a study of caged mink, Meagher and Mason [51] found that, contrary to their predictions, repetitive scratching at the cage wall (possibly analogous to the gnawing at the cage corner by the *Peromyscus* ) was not associated with measures of boredom (interest in both aversive and rewarding stimuli), and stereotypic locomotor behavior (possibly analogous to the flipping behavior) was inversely correlated with these measures of boredom. When behaviors (or morphological and physiological changes) are observed that are presumed to reflect stress and possibly be maladaptive, it might be beneficial to test changes to the environment that could reduce the trait [52,53] rather than trying to select against the trait without changing the environment that triggered its expression.

It is risky to presume that we know enough about what is adaptive (including all possible indirect and correlated effects) to be able to genetically select against maladaptive traits and for more rapid adaptation to the novel environment [20,54]. At a minimum, we suggest that before artificial selection on behavior is practiced in an attempt to improve fitness in captivity, experiments be conducted to document which behaviors do correlate with good reproduction (e.g., [55]) and to confirm that there are no unintended and undesirable correlated responses in other traits. Given that natural selection would be strong in the captive environment whenever genetic adaptation is necessary to allow maintenance of the population, it would seem that a more cautious and likely more effective approach would be to let response to that selection provide the necessary adaptation to captivity. This could be accomplished simply by breeding as many of the animals as possible, as should be done anyway if there is risk of losing the population.

## Conclusions

Evolutionary change in *Peromyscus* *leucopus* mice propagated in a captive environment occurred rapidly, with a greater proportion of pairs breeding and an acceleration of breeding occurring over the first 10 generations in randomly bred experimental populations. Similar rates of adaptation to captivity occurred when pairs were chosen by minimizing population mean kinship, and thereby maximizing retention of gene diversity. Both random breeding and minimizing kinship led also to increases in repetitive flipping behavior, which had been hypothesized to be a maladapted response to the captive environment but was found instead to be strongly positively correlated with fitness in captivity.

Artificial selection against flipping and gnawing behavior eliminated flipping, but with the consequence that reproduction declined, the populations become more rapidly inbred, and one of two selected populations failed to reproduce after 9 generations. This unexpected outcome indicates that it can be difficult and even counter-productive to attempt to control evolutionary adaptation to a novel environment through artificial selection.

The mean kinship minimization achieved a 40% increase in N_e_, slowed the accumulation of inbreeding, and resulted in less divergence between replicate populations than was observed under random breeding. However, that breeding protocol had little effect on the rate of adaptation to captivity. Small decreases in some components of reproductive success in all populations could have been due to inbreeding depression and were reversed by outcrossing between previously isolated populations.

The rapid changes observed suggest that laboratory stocks and other populations that are generations removed from the wild might not be good models for studies of species typical reproductive patterns and behavior. It is not known what physiological changes may also have occurred in response to the captive environment, but there is no reason to believe that other traits did not similarly diverge rapidly from the phenotypes that had evolved in the wild.
